# Patient-Reported Outcomes Measurement in Radiation Oncology: Interpretation of Individual Scores and Change over Time in Clinical Practice

**DOI:** 10.3390/curroncol29050251

**Published:** 2022-04-27

**Authors:** Jae-Yung Kwon, Lara Russell, Theresa Coles, Robert J. Klaassen, Kara Schick-Makaroff, Kathryn M. Sibley, Sandra A. Mitchell, Richard Sawatzky

**Affiliations:** 1 School of Nursing, University of Victoria, Victoria, BC V8P 5C2, Canada; 2 Institute on Aging and Lifelong Health, Victoria, BC V8N 5C2, Canada; 3 School of Nursing, Trinity Western University, Langley, BC V2Y 1Y1, Canada; lara.russell@twu.ca (L.R.); rick.sawatzky@twu.ca (R.S.); 4 Department of Population Health Sciences, Duke University School of Medicine, Durham, NC 27701, USA; theresa.coles@duke.edu; 5 Division of Hematology/Oncology, Department of Pediatrics, Children’s Hospital of Eastern Ontario Research Institute, University of Ottawa, Ottawa, ON K1N 6N5, Canada; rklaassen@cheo.on.ca; 6 Faculty of Nursing, University of Alberta, Edmonton, AB T6G 1C9, Canada; kara.schickmakaroff@ualberta.ca; 7 Department of Community Health Sciences, University of Manitoba, Winnipeg, MB R3E 0W3, Canada; kathryn.sibley@umanitoba.ca; 8 George and Fay Yee Centre for Healthcare Innovation, University of Manitoba, Winnipeg, MB R3E 0W3, Canada; 9 Outcomes Research Branch, Healthcare Delivery Research Program, Division of Cancer Control and Population Sciences, National Cancer Institute, Rockville, MD 20850, USA; sandra.mitchell@nih.gov; 10 Evaluation and Outcome Sciences, Providence Health Care Research Institute, Vancouver, BC V6Z 2K5, Canada; 11 Sahlgrenska Academy, University of Gothenburg, 40530 Gothenburg, Sweden

**Keywords:** patient-reported outcome measures, differential item functioning, response shift

## Abstract

Tools for measuring patients’ perceived health and quality of life, such as patient-reported outcome measures (PROMs), inform clinical decisions for patients requiring radiation therapy. However, there may be inconsistencies in how patients interpret and respond to PROMs due to cultural, environmental, personal, or experiential factors. Differential item functioning (DIF) and response shift (RS) refer to differences in the meaning of PROMs between patients or over time (respectively). DIF and RS can threaten the accurate interpretation and use of PROMs, potentially resulting in erroneous conclusions about effectiveness, and flawed individual-level clinical decision-making. Given the empirical evidence of DIF and RS, we aim to review clinical implications and solutions for addressing DIF and RS by providing vignettes from collaborative examinations with workshop participants, as well as the literature. By making these methodological concepts accessible and relevant, for practice, clinicians may feel more confident to ask clarifying questions of patients when PROM scores and the contextual patient information do not align. PROM scores need to be interpreted via dialogue with the patient to avoid misinterpretation due to DIF and RS, which could diminish patient–clinician communication and impede shared decision-making. This work is part of an interdisciplinary knowledge translation initiative focused on the interpretation of PROM scores by clinically-oriented audiences.

## 1. Introduction

Increasingly, clinicians, payers, and regulators look to patient-reported outcome measures (or PROMs, which allow patients to directly report on their health status, quality of life, symptoms, and functional status) to understand how patients are affected by their cancer and its treatments [1,2]. However, despite widespread acknowledgement that PROMs provide valuable data, challenges exist in the interpretation of PROM scores in clinical practice for diverse populations [3]. There is evidence to suggest that clinicians either over- or underestimate the symptom burden and adverse effects in traditional monitoring, which involves clinicians taking a patient’s history and conducting medical assessments (e.g., imaging and blood tests) [4,5]. For example, patients undergoing radiation therapy have high levels of anxiety and depression that is often under-detected and undertreated [6]. As cancer care becomes more complex, compounded by advances in diagnostic and treatment strategies (e.g., surgery, chemotherapy, immunotherapy, radiation therapy, or a combination of these), and multifaceted clinical presentations [7,8,9], it is important to capture patients’ own perceptions of their treatment and its impact to facilitate improved patient–clinician communication and shared decision-making [10,11,12]. Unfortunately, without an awareness of biases in the interpretation of PROM scores, we risk diminishing the accuracy and utility of PROMs in shaping the goals of care, and in guiding individual treatment decisions. Specifically, potential measurement biases occur when PROM scores are not directly comparable between different people or over time due to the presence of differential item functioning or response shift.

Differential item functioning (DIF) occurs when the same PROM items do not equivalently reflect the outcome being measured when used with different people [13]. For example, two patients who actually have the same level of pain may provide different PROM scores for pain severity. These differences may be due to different expectations, different meanings, different culture, personality or coping style, or different impact or degree of interference the pain causes for daily activities. For example, the Western perspective may primarily view pain as an external disturbance that interferes with everyday life, whereas other cultures may view pain as being part of one’s life journey [14], which may influence how patients respond to PROM items. In addition, response shift (RS) occurs whenever observed change (e.g., responses to PROM items) is not the same as target change (i.e., change we want to measure) [15], resulting in measurements at two or more time points not being comparable. For example, a patient may interpret and respond to a PROM item for measuring pain differently before and after undergoing treatment due to a “shift” in their internal frame of reference by which they assess their pain. Retrospectively, they may regard their pain before treatment as being less severe. In this situation, the difference between pain scores before and after treatment (observed change) does not accurately reflect the difference in level of pain that we wish to measure (target change). 

Studies have shown that ignoring DIF and RS of PROMs can lead to measurement biases and resultant misinterpretations when individual PROM scores do not have the same meaning for different socio-demographic groups or over time [16,17]. DIF or RS occur when the outcome is not measured consistently between groups or across time (in technical terms, the statistical measurement model is *not invariant* between groups or across different points in time). There are three different types of measurement invariance that can result in DIF and RS (see Table 1 for definitions and implications). 

DIF and RS have historically been studied at the group level, and little work has been done to translate methodological understandings of DIF and RS for individual-level PROM score interpretation. In clinical practice, both clinicians and patients would directly benefit from greater awareness of potential measurement biases so that they can draw more accurate conclusions based on PROM scores. To address this gap, our research team co-developed educational resources with researchers, analysts, and clinicians to inform them about DIF and RS, creating methods to address them in practice, and improve the interpretation of PROMs.

### 1.1. Purpose and Context

The purpose of this paper is to review the clinical implications and potential approaches to anticipate and accommodate possible DIF and RS when interpreting PROM scores at the individual level. In so doing, we intend to raise awareness of DIF and RS and their implications, and to increase the ability of clinicians to identify and address DIF and RS in the interpretation of PROMs to facilitate patient–clinician communication and shared decision-making.

Examples in this manuscript are based on a knowledge translation (KT) initiative designed to facilitate wider uptake of knowledge for interpreting and analyzing PROM scores, aimed at clinicians who can directly address DIF and RS in their practice. The initiative specifically involved developing two educational resources about the individual-level interpretation of PROM scores for healthcare decision-making (a webinar and a learning module).

### 1.2. Approach

The results presented were informed by discussions with members of the Clinical Practice and Response Shift Special Interest Groups at the International Society of Quality of Life (ISOQOL) as part of the KT initiative. All members were invited through the ISOQOL listserv, and include researchers and clinicians in various practices, such as family medicine, neurology, and cancer (*n* = 12), who shared a common interest in improving the capture and interpretation of PROMs to support individual-level decisions in clinical practice settings.

We conducted four online workshops of 1.5 h each to obtain input on the content and format of the educational resources (see project website of the developed resources: healthyqol.com/methods). Workshop participants were initially introduced to the concepts of DIF and RS with introductory videos (see link: https://youtu.be/LZrgSRU-psQ, accessed on25 March 2022) comprising presentations and clinical examples in order to make these concepts more accessible to clinicians. Emerging ideas were then discussed for further development in subsequent sessions. For example, questions for discussion included: What should we be aware of when developing educational resources for clinicians? What type of case studies (real-life examples) would help to identify DIF/RS in practice? What interactive learning modalities (e.g., video, graphics, quizzes) might be most effective for clinicians? During these discussions, a hypothetical patient case example of “Bill” was developed to make learning these complex methodological concepts of DIF and RS more engaging and clinically applicable. Extrapolation of the vignettes were guided by a collaborative examination of the discussions in the online workshops, focusing on the type of dialogue between Bill and a clinician, as well as the literature on DIF and RS. Through this review, we identified important aspects of DIF and RS to further explore and identify in the dialogue. For example, we explored differences in meaning that the PROM scores may have for Bill compared to others depending on life situations. In addition, the research team and the workshop participants reviewed the vignettes. It is important to note that the following vignettes are intended to inform clinicians on the use of PROMs, but are not specifically meant to imply that the interaction and leading questions are the “right” ones to reveal and further probe potential sources of DIF and RS.

To make the methodological content of these insights more accessible and relevant for practice, we created the following hypothetical case example of a patient named “Bill”:

“Bill” is a 70-year-old from a rural community who recently lost his wife and is also coping with multiple chronic illnesses. After completing radiation to treat stage 3 lung cancer, he follows up with his radiation oncologist, and completes PROMs during clinic visits. The oncologist uses the PROM responses to track changes in Bill’s health status over time and uses them when comparing other patients receiving similar treatment during clinical interactions. However, aware of the possible influences of DIF or RS, the clinician interprets PROM scores in dialogue with Bill to arrive at a more accurate picture of his health status, and tailors the approach to Bill’s care and treatment accordingly.

## 2. Results

### 2.1. Types of Differential Item Functioning (DIF)

The first consideration in the interpretation of PROMs pertains to DIF, which occurs when the same questionnaire items do not equivalently reflect the outcome being measured when applied to different people [13]. DIF leads to measurement biases in three ways:

(1) Lack of **scalar invariance**: individuals interpret the items and/or response scales differently from other people.
(2) Lack of **metric invariance**: individuals assigning different meanings to items used for measuring a construct (e.g., health).
(3) Lack of **configural invariance**: individuals not defining the construct (e.g., health) the same as other people.

**Figure 1:**
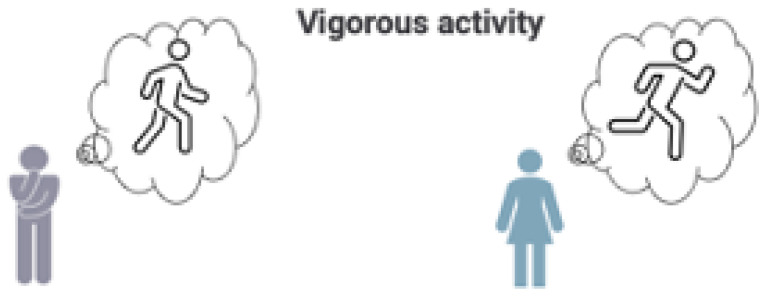
Bill (in grey) interprets items differently compared to others (lack of scalar invariance).

Consider first an example of the lack of scalar invariance, illustrated in Figure 1 and the dialogue below:

Clinician: Bill, you rated vigorous activity as moderately difficult. Many of my other patients who are very active think of running when they are imagining a vigorous activity. Is that what you were thinking of when rating vigorous activity as moderately difficult?

Bill: Ah, I see what you mean. For me, vigorous activity does not mean running, but rather, being able to walk around my neighborhood without losing my breath.

This example illustrates how DIF can manifest as a lack of scalar invariance. Bill was interpreting vigorous activity as walking without becoming short of breath, which is different from how others may have interpreted the question (i.e., running). If scalar invariance were ignored, Bill could have received more aggressive cancer treatment that would likely reduce his physical function abilities further. Patients’ physical function pre-treatment may be critical to healthy recovery post-treatment, thus clarifying patient-reported functioning as critical when making treatment decisions. To address potential misinterpretation in the meaning of PROM items for different groups of people due to cultural, demographic, life circumstances, and/or different health experiences, and to better manage expectations and goals for treatment, clinicians may need to ask for clarification regarding how patients understand words describing levels of physical activity such as “vigorous.”

**Figure 2:**
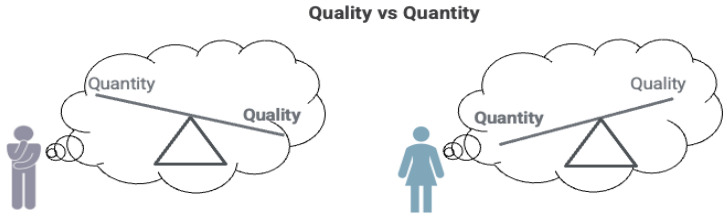
Bill (in grey) values underlying health domains differently compared to others (lack of metric invariance).

A lack of metric invariance leads to different kinds of issues, as illustrated in Figure 2 and the dialogue below:

Clinician: Bill, you rated the “worrying about dying” item as “very much”, and this raises an important question. In answering this question, are you concerned about the quality of your life or the length of your life, or both? Let me put this in the context of cancer treatment: some patients choose a treatment with a goal to prolong their life, while others are more concerned with potential side effects and complications that could affect their quality of life.

Bill: I would rather live a shorter life where I could do more with better quality of life than take a chance on treatment that may extend my life, but result in significant complications.

Clinician: That’s important to know. Let’s talk more about different treatment options and their potential side effects that could affect your quality of life.

In this discussion, Bill and his clinician are exploring how DIF can manifest as a lack of metric invariance. Bill’s initial answers on the PROM were ambiguous with regard to whether he valued quality of life over quantity. If the clinician substituted his own interpretation of Bill’s answers and imposed lack of metric invariance on his responses, he may have recommended surgery that Bill did not actually want. Asking follow-up questions helped Bill clarify an important distinction that he values quality of life as being more important compared to others who may prefer more years of life. Clinicians can use such information derived from PROM clarification in treatment decisions, providing information regarding different treatment options and the side effects of each in the context of Bill’s emphasis on quality of life.

**Figure 3:**
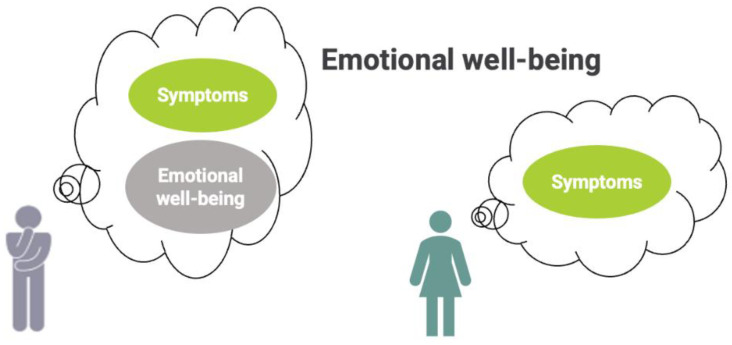
Bill (in grey) includes different factors in his definition of health compared to others (lack of configural invariance).

A final instance of how DIF can manifest as lack of configural invariance is illustrated in Figure 3 and the dialogue below:

Clinician: You rated your overall health rating as being quite low, even though you report no symptoms. Can you tell me more about why you’re not happy with your health despite not having any symptoms?

Bill: Well, I don’t have a lot of physical symptoms, but I’m anxious about my cancer diagnosis and sometimes feel down.

Clinician: So, in your case, feeling healthy includes your emotional well-being, and being worried about your diagnosis makes you feel discouraged. That’s understandable. Let me give you some information on resources to help address your concerns about your cancer treatment and improve your overall health.

This example of DIF is a case of lack of configural invariance because, unlike other people whose health construct is limited to just their symptoms or how they function physically in daily life, Bill’s underlying definition of health includes his emotional well-being. The lack of configural invariance seen in his answers should not be disregarded with the erroneous assumption that he is contradicting himself; clinicians should instead see what might appear to be paradoxical responses on a PROM as an opportunity to understand how different patients define their own health. If a lack of configural invariance was not taken into account, Bill’s concerns could have been ignored, further exacerbating his symptoms and reducing his overall quality of life. Awareness of possible forms of DIF and probing with thoughtful questions, as the dialogue shows, can help to improve the interpretation of PROMs at the individual level, thereby allowing for tailored disease-related treatment, goal concordant care, and optimized provision of supportive, palliative, and rehabilitative therapies.

### 2.2. Types of Response Shift (RS)

The second consideration in the interpretation of PROMs pertains to RS, which is a form of longitudinal DIF when a discrepancy between observed (e.g., change in PROM scores) and target change (e.g., change in health) occurs [15]. This discrepancy leads to measurement biases in three ways:

(1) **Recalibration**: change in internal standards of measurement by which people interpret items and response scales.
(2) **Reprioritization**: change in the relative importance (i.e., assigned meaning) of domains or items.
(3) **Reconceptualization**: change in the definition of the target construct.

**Figure 4:**
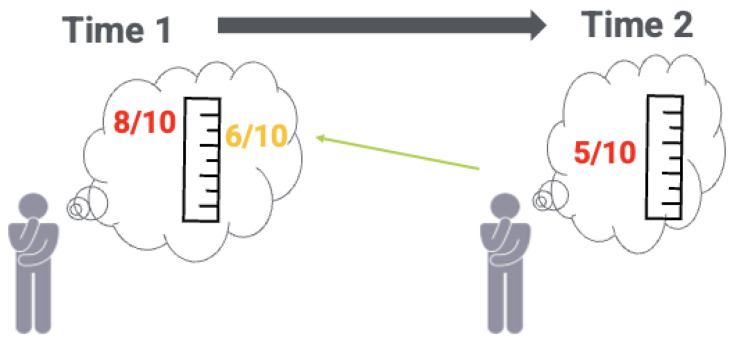
Bill adjusts his perspective regarding his health over time (recalibration).

The first kind of RS is seen in the recalibration scenario below (See Figure 4 and dialogue):

Clinician: Looking at your PROM scores, I see that you rated your overall pain as an 8 before your radiation therapy, and a 5 afterwards. It’s good that you feel your pain has improved, but you’ve mentioned to me a few times that you’re still experiencing some issues. Can you explain why your pain score improved by 3 points, even though you have discomfort?

Bill: Actually, looking at my “before” and “after” scores side by side and thinking back to how I felt before, I realize that 8/10 was too high. I really feel that 5/10 is a good number for how I feel now, but comparing that to how I felt before, I now think I should have rated my overall pain back then as 6/10.

Clinician: Thanks for explaining. While it seems like your new score suggests that things are getting better, your new perspective on your pain now relative to your pain before treatment improves my understanding of your recovery process. Would you like a referral to a pain specialist to better manage your pain? Is there anything else we could address to improve your overall pain right now?

Bill: You know, that would be great. I really am grateful that I can still walk, but the pain doesn’t seem to be lessening over time.

This example shows a situation where Bill, in trying to come to terms with his condition, reported improvement in overall pain after radiation therapy, even though he was still having some discomfort. If response shifts involving recalibration are not identified and addressed, the impact of treatments as measured by PROMs may be over- or underestimated. For clinicians, it is important to work with patients to understand what is happening contextually and acknowledge potential changes to their frames of reference that may occur over time. Doing so can help patients in their health journey, and meet their needs as these evolve over time.

**Figure 5:**
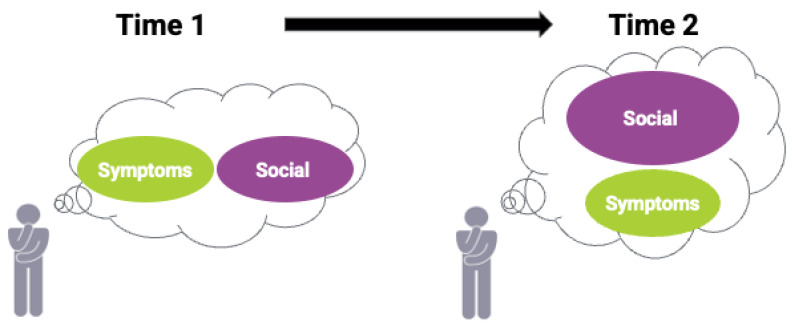
Bill changes which health domains matter most over time (reprioritization).

A second form of RS can occur in the case of reprioritization (See Figure 5 and dialogue):

Clinician: I noticed that your latest PROM scores regarding social well-being are different from before. Can you explain your shift in thinking?

Bill: While I valued the time with family and friends before this intense radiation treatment, I thought being cancer-free was equally important to me. But after treatment, I’ve come to realize that while I still want to be cancer-free, spending quality time with family and friends is much more important and wanted to convey that in my latest PROM.

Clinician: That’s good to know. Based on your changed priority, does the current treatment seem to negatively impact your social functioning?

This scenario shows how patients with cancer, such as Bill, can reprioritize what matters to them over the course of their treatment. Though Bill still values being “cancer-free”, after radiation therapy, he has come to value social function more. Recognizing response shifts over time is important, as patients may be prescribed treatment that could (in Bill’s case) have adverse effects on their social function, whereas before such treatments, might seem desirable given his interest in being cancer-free. Clinicians need to be attuned to the evolution of PROM scores over time to ensure that treatment and its side effects do not impact patients negatively if they reprioritize their health domains.

**Figure 6:**
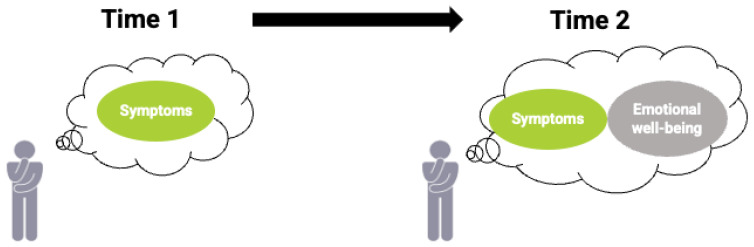
Bill has redefined what health means to him by including emotional well-being (reconceptualization).

A final RS scenario involves reconceptualization (See Figure 6 and dialogue):

Clinician: Looking at your health questionnaire from before and after radiation therapy, it looks like your overall health scores have improved, even though you are still reporting some pain and depression. Can you explain what has changed for you after treatment?

Bill: There are still symptoms, but I’ve started to meditate, which has helped to reduce my anxiety and improve my sleep. The improvement in my symptom scores reflect that. Thanks for giving me the chance to explain my scores on the questionnaire.

Clinician: You’re welcome. I asked because otherwise it would seem like you were having side effects of treatment and I might have even suggested a different path forward.

Seemingly inconsistent or puzzling scores on PROMs oftentimes reflect response shift—in this instance, reconceptualization because Bill has redefined his health after treatment to include his emotional well-being. When such response shifts are ignored, clinicians may interpret the higher (or lower) PROM scores to the effects of cancer-treatment itself, which could have implications for continuing the current cancer-directed treatment. All three RS scenarios highlight the importance of interpreting PROMs over time in dialogue with patients to improve the accuracy of our interpretations of change in health status, and as a basis for shared decisions and alignment of goals with current priorities with individual needs and experiences. 

## 3. Discussion

In this paper, we applied the concepts of DIF and RS to clinical practice settings when interpreting individual PROM scores, and illustrated how to incorporate individual PROM reports into an approach that serves to promote communication, shared decision-making, and a mutual understanding of progress towards goals. To avoid measurement biases, it is critical that clinicians who are using PROMs take into account the possibility that different patients have unique interpretations of PROM questions and the response choices, and that their interpretation may shift over time. This was illustrated through the examples of dialogue with our hypothetical patient “Bill”, which were based on our KT project that addresses an important gap in the translation of complex methodological topics on PROMs by exemplifying an approach to be more accessible to clinical audiences. 

In radiation oncology, the consideration of DIF and RS is particularly important to monitor the impact of treatment on the outcomes of diverse individuals, since the effects of radiation therapy on symptoms and quality of life can only be truly assessed from the patients’ point of view. In cancer care, there is a strong cultural belief that having an optimistic attitude can improve patient outcomes [19]. This belief can potentially influence patients’ responses to PROM items, leading to instances of DIF and RS that result in higher PROM scores despite the severity of symptoms. It is, therefore, important for clinicians to consider the contextual information they already know about their patients. When PROM scores and the contextual information do not align, it is important to ask clarifying questions such as those in the sample dialogues in order to better understand changes in health based on patients’ PROM scores, thereby providing a sound basis for shared decision-making. At the same time, if the cancer is chronic and cannot be cured, patients can be helped by changing their frame of reference through a process of adaptation (resulting in recalibration, reprioritization RS). In these situations, the occurrence of RS can result from a better alignment of patient preferences with the goals of treatment, as is the case in most patient education [20] and psychological treatments (e.g., cognitive behavioral therapy) [21].

### 3.1. Implications for Practice

The implication in practice is that PROM scores need to be interpreted via dialogue with the patient to avoid misinterpretation due to DIF and RS. For example, when a choice needs to be made between different treatment options, patients’ cultural, environmental, personal, or experiential factors need to be taken into account, in a process of shared decision-making. Thus, a key recommendation for clinicians is not to take PROM scores and change over time at face value, but rather, as is the case with “white coat hypertension” [22], to use them as a starting point for conversations with the patient to prevent misinterpretation that could diminish communication or impede shared decision-making. We argue that the utility of PROMs is contingent on their sound interpretation by taking into account measurement biases such as DIF and RS; otherwise, potential negative consequences can result that can misalign treatment goals to the needs of the patient. Feedback from clinicians on potential interpretational differences among patients could guide future qualitative research into DIF and RS regarding specific hypotheses about certain PROM items (e.g., the meaning of “vigorous” for certain subgroups). DIF and RS at the individual level deserves greater attention, as there is continued progress towards the inclusion of PROMs to improve communication and inform shared decision-making in clinical practice.

### 3.2. Conclusions

Given the novelty of considering DIF and RS in the context of healthcare decision-making, we need empirical studies to examine under what circumstances DIF and RS affect the types of decisions made in practice. Such studies may also teach clinicians how DIF and RS relate to other known biases due to, for example, social desirability bias. With more empirical data available, we expect to better understand how to account for DIF and RS for more accurate conclusions about the meaning of PROM scores to facilitate improved patient–clinician communication and shared decision-making. We hope that increasing awareness of the often-neglected influence of DIF and RS in the interpretation of PROM scores at the individual level will stimulate further discussion with clinicians in radiation oncology and improvements in PROM design, as well as the quality of healthcare decision-making by accurately representing the health and quality of life concerns of individual patients.

## Figures and Tables

**Table 1:** Implications of different types of differential item functioning and response shift.

DIF/RS	Oncology Example	Types	Examples of Implications for Clinical Practice
DIF: Two patients who have the same level of health may report different PROM scores (e.g., due to cultural, environmental, personal, or experiential differences)	For items of physical and emotional functioning, scalar DIF relative to sex were observed, where males were more likely to endorse items with intensive physical activities and irritability than females [18]	Lack of scalar invariance (interpret items and response scales differently from others)	If this type of DIF is ignored, the decision to address side effects of treatment (e.g., worsening physical function, increased irritability) may be overlooked for males
	Relative to Caucasian and Japanese groups, items related to physical, cognitive and social functioning, nausea and vomiting, and financial difficulties exhibited DIF for Filipinos. On these items, Filipinos exhibited either higher or lower quality of life (QoL) scores, even though their overall QoL was the same [17]	Lack of metric invariance (assign different meanings to items used for measuring health)	If this type of DIF is ignored, Filipino patients’ concerns about impaired quality of life may not be addressed because scores do not reflect those aspects that are most problematic when given cancer treatment
		Lack of configural invariance (define health differently from others)	If this type of DIF is ignored, it will be more difficult for clinicians to prioritize interventions based on how patients define aspects of their health
RS: A patient has changes in their health level, but report the same PROM scores over time	Majority of patients with bone metastases with palliative radiation therapy (73%) had reduction in pain scores, but response shift resulted in no changes in overall pain score [16]	Recalibration (change in measurement standards)	If this type of RS is ignored, the impact of treatments based on PROMs may either be over or under-estimated
		Reprioritization (change in relative importance of items)	If this type of RS is ignored, patients may be prescribed treatment that could have adverse effects on their social function, which may be more important than being cancer-free
		Reconceptualization (change in definition of construct)	If this type of RS is ignored, clinicians may attribute higher pain scores to the treatment itself, and may, therefore, no longer continue the treatment
